# Automated Support Generation for Fixed Partial Dentures and Impact of Bone Loss, Bone Quality and Support Types: Parametric Cad and Finite Element Analysis

**DOI:** 10.3390/dj12120394

**Published:** 2024-12-04

**Authors:** Hassen Jemaa, Michael Eisenburger, Andreas Greuling

**Affiliations:** Department of Prosthetic Dentistry and Biomedical Materials Science, Hannover Medical School, 30625 Hannover, Germany; jemaa.hassen@mh-hannover.de (H.J.); eisenburger.michael@mh-hannover.de (M.E.)

**Keywords:** bone resorption, bone density, fixed partial denture, finite element analysis, computer-aided design

## Abstract

**Background:** This study aimed to develop an algorithm for modelling tooth–tooth or implant–implant support configurations for a given 4-unit fixed partial denture (FPD). **Methods**: The algorithm was implemented in Rhinoceros/Grasshopper to automatically generate geometries with varying bone loss (0 mm to 3 mm), support type (tooth–tooth and implant–implant support) and bone quality (D1 to D4) for a 4-unit FPD. Afterward, a finite element analysis was carried out with a load applied to the central connector of the FPD. Stresses in the FPD and the bone around the support were analysed. **Results**: The results indicated that stresses in the bone were influenced by both the depth of bone loss and bone quality across both support types. The maximum stress in the tested FPD models for tooth–tooth support was not significantly influenced by bone quality; instead, the stress peaks were primarily influenced by the depth of bone loss. For D1 bone, the stresses in FPD and bone increased by 4.79% and 8.86%, respectively, for tooth–tooth support and about 32.67% and 100.96%, respectively, for implant–implant support. **Conclusions**: The proposed algorithm allows for the automatic, parametrised generation of support for 4-unit fixed partial dentures, which can be used to predict the effect of bone loss and bone quality on stresses for patient-specific geometries. The optimal treatment for support type should be adapted to the patient’s specific needs to ensure long-term stability. A decision support involving automatic modelling, e.g., of support, and simulation might improve clinical treatment planning from a long-term perspective.

## 1. Introduction

All-ceramic restorations for FDPs in the posterior region have achieved satisfactory results in terms of durability in long-term clinical studies [1,2]. However, technical complications like fracture of the framework and/or veneering or de-cementation sometimes still occur [2].

The survival rate of a ceramic-based FPD on natural teeth or implants depends on various factors, such as material properties [3], cross-sectional area of connectors [4], bone support type [5], bone quality [6] and geometric dimensions of the implant system [7]. Chandranaik et al. reported in a clinical survey of 450 FPDs that mechanical failures were the primary cause for FPD failures, accounting for 51.1%, followed by biological failures at 33.3% and aesthetic failures at 11.5% [8]. Campaner et al. studied the impact of polymeric restorative materials on stress distribution in posterior fixed partial dentures via finite element analysis (FEA) and found that the materials had different effects. Resin composite was found to reduce the stresses on the cement layer, while acrylic resin attenuated the stresses in the connector region [9]. A study by Zarnpelis et al. looked into the angle of support inclination and found that tilting the support structure for FPD did not lead to higher bone stress peaks compared to vertically placed supports [10]. Larsson et al. found that the design of the connector affects the fracture strength of fixed partial dentures [11]. Several studies have reported on the survival rates of reconstructions with different support types and the complications that occur during failure [12,13,14,15].

Besides the mentioned factors, periodontal or peri-implant bone loss can also affect the survival of FPDs. This aspect has not been extensively studied. One possible way to obtain a prognosis for the effect of expected bone loss on stresses in the FPD is the application of the finite element method. As already stated in previous work [16], the finite element method is currently not used for routine clinical decision support for dental implants in patient-specific cases. This is because the cost of creating CAD models and performing simulations is too high, as it requires individual work and the degree of automation is not sufficient yet. In previous work, a method for the semi-automated generation of bone loss defects around dental implants using parametric modelling was developed [16]. Parametric modelling has also been used elsewhere in the literature for modelling a human hand [17] or to remodel the connectors for a FPD [18]. To the best of the authors’ knowledge, an automated generation of a FPD support has not yet been described in the literature, although it is an essential component when aiming at fully automated model generation.

The main goal of this study was to develop an algorithm for parametric generation of a support for a given 4-unit fixed partial denture. To the best of the authors’ knowledge, a similar algorithm is not present in the literature. The proposed parametric algorithm was used to investigate the influence of bone loss and bone quality on stresses in a FPD and bone for two types of support: implant–implant support and tooth–tooth support. It is hypothesised that increasing bone loss leads to increasing stresses in bone and FPD due to a lack of support. Furthermore, a bone quality with lesser bone density is also expected to lead to higher stresses due to higher mobility.

## 2. Materials and Methods

In this study, parametric algorithms for the automatic generation of a support for 4-unit fixed partial dentures were developed. These algorithms were implemented in Rhinoceros/Grasshopper v7 (Robert McNeel & Associates, Seattle, WA, USA), a tool that allows for complex 3D modelling. The flexibility and user-friendly platform of Grasshopper make it particularly well suited for automating repetitive modelling tasks through a visual programming interface. The methodology of the proposed algorithm can be adapted for implementation in other modelling software. While the basic steps would be similar to those described in this manuscript, this might require rewriting Grasshopper-specific functions if similar ones are not available in the alternative modelling software. Two different types of support, tooth support and implant support, were addressed. 

The implementation was used to investigate the effect of bone loss and bone quality on a 4-unit FPD with different kinds of support using a finite element analysis (FEA). The FEA was performed using ANSYS workbench 2022R1 (ANSYS Inc., Canonsburg, PA, USA). The geometry of the 4-unit FPD was acquired using an optical scanner (ATOS II SO, GOM GmbH, Braunschweig, Germany) and a master model already used in previous studies [18,19,20].

The steps required for 3D parametric modelling and finite element analysis are described in detail in the following sections. The abbreviations in subsequent sections denote *P*: points, *L*: lines, *C*: curves, *E*: planes, *M*: meshes, *B*: bounding boxes, *f*: faces, $\vec{v}$: vectors, *S*: coordinate systems and *d*: distances or diameters, respectively.

### 2.1. CAD Modelling

#### 2.1.1. Manual Preprocessing

**Figure 1:**
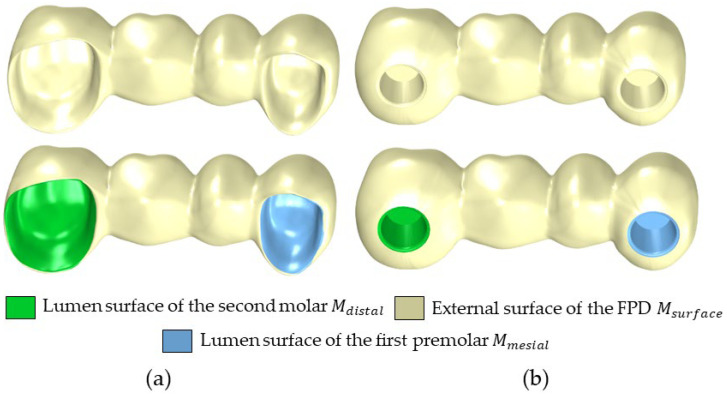
Overview of the 4-unit FPD. Division of the mesh into three meshes (*M_surface_*, *M_distal_* and *M_mesial_*) for further processing. (**a**) FPD for tooth–tooth support. (**b**) FPD for implant–implant support.

The mesh for two 4-unit FPDs, one variant for tooth support and one variant for implant support, was obtained using the aforementioned optical scanner; see Figure 1a,b. Each mesh was imported into Meshmixer (Autodesk Inc., San Francisco, CA, USA). In Meshmixer, each mesh was manually divided into three parts: the lumen distal *M_distal_* (second molar), the lumen mesial *M_mesial_* (first premolar) and the external surface *M_surface_*, as illustrated in Figure 1. Although this step could, in principle, be automated, this was outside the scope of the current work. This is because the long-term goal is to generate the whole FPD automatically, which will make this step unnecessary, as the mesh division/labelling will be part of that process. The three parts *M_surface_*, *M_distal_* and *M_mesial_* were exported into a single STL file for further processing. 

#### 2.1.2. Automatic Generation of FPD Support

**Figure 2:**
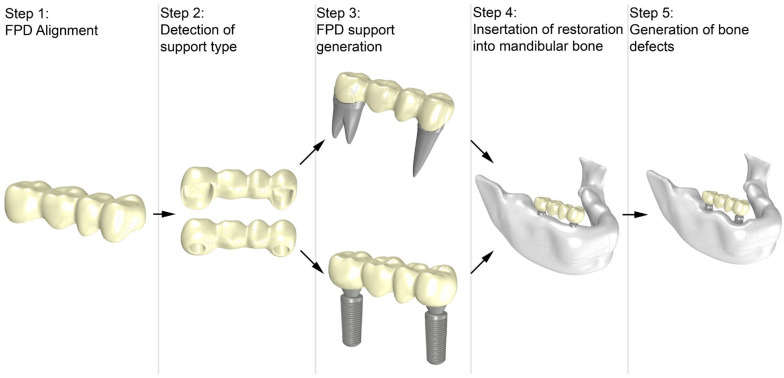
Workflow for the automatic generation of a support for 4-unit FPD, insertion into bone and generation of bone defects.

The STL file from the previous step, which includes the three parts: *M_surface_*, *M_distal_* and *M_mesial_*, was loaded into Rhinoceros/Grasshopper, where an automated support generation for the FPD was performed. An overview of the whole process is depicted in Figure 2. Firstly, the STL file of the 4-unit FPD was imported into the software and subsequently aligned. A geometric analysis of the lumen surfaces was performed to determine the support type required to generate the geometries. The support was then generated, including cement, tooth and periodontal ligament for tooth-supported FPDs and cement, abutment, implant and screw for implant-supported FPDs, all based on user-defined parameters. The FPD and its support were automatically placed in the mandibular bone, and bone loss defects were generated around the implants. Detailed descriptions of each step are provided in the following sections.

**FPD alignment (step 1)**

**Figure 3:**
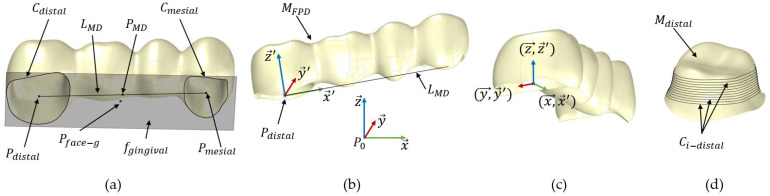
Illustration of the FPD alignment sub-steps for a tooth supported FPD. The implant supported FPD was handled the same way. (**a**) Basal view of FPD. (**b**) Side view with global *S_global_* (*P_0_*,$\vec{x},\;\vec{y},\;\vec{z}$)) and local coordinate system (*S_FPD_* (${\vec{x}}^{\prime },\;{\vec{y}}^{\prime },\;{\vec{z}}^{\prime }$)). (**c**) Alignment of coordinate systems *S_FPD_* with *S_global_*. (**d**) The intersection curves *C_i-distal_* used for support type detection (step 2).

After importing the FPD geometry into Rhinoceros/Grasshopper, the alignment of the FPD to the principal coordinate system *S_global_* (*P_0_*,$\vec{x},\vec{y},\vec{z}$) was achieved through the following steps:

(1) Merge *M_mesial_*, *M_distal_* and *M_surface_* into a single mesh, *M_FPD_*.
(2) Extract the boundary curves *C_distal_* and *C_mesial_* from the open surfaces *M_distal_* and *M_mesial_* using the *naked edge curve* function in Grasshopper, respectively; see Figure 3a. These boundary curves represent the open curves of the surfaces, which prevent the mesh from being closed.
(3) Find the centre points *P_distal_* and *P_mesial_* for the curves *C_distal_* and *C_mesial_*.
(4) Create the line *L_MD_* between *P_distal_* and *P_mesial_* and compute its midpoint *P_MD_*.
(5) Compute the smallest bounding box, *B_min_*, for the mesh *M_FPD_* using the *bounding box* function in Grasshopper.
(6) Deconstruct the bounding box *B_min_* into 6 faces, *f_i_*, and compute the centre point, *P_face-i_*, for each face.
(7) Identify the gingival face, *f_gingival_*, which centre point is closest to the point *P_MD_*; see Figure 3a.
(8) Create a plane, *E_FPD_*, parallel to *f_gingival_* at *P_distal_*.
(9) Define a local coordinate system for *M_FPD_*, denoted as *S_FPD_* (${\vec{x}}^{\prime },\;{\vec{y}}^{\prime },\;{\vec{z}}^{\prime }$). This coordinate system is centred at *P_distal_*. The *z*-axis (${\vec{z}}^{\prime }$) of *S_FPD_* is set to be perpendicular to *E_FPD_* at *P_distal_*. The *x*-axis (${\vec{x}}^{\prime }$) is defined to align with the line *L_MD_*, and the *y*-axis (${\vec{y}}^{\prime }$) is constructed to be orthogonal to both $x^{\prime }$ and ${\vec{z}}^{\prime }$; see Figure 3b.
(10) Translate *M_FPD_* so its local coordinate system’s origin *P_distal_* matches the global coordinate system’s origin *P_0_* via the Grasshopper function *move to point*.
(11) Rotate *M_FPD_* to align its local axes *S_FPD_* (${\vec{x}}^{\prime },\;{\vec{y}}^{\prime },\;{\vec{z}}^{\prime }$) with the global coordinate system’s axes *S_global_* ($P_{0},\vec{x},\vec{y},\;\vec{z}$) using the function *Orient*; see Figure 3c.

**Detection of support type (step 2)**

In this step, the FPD support type was detected and assigned to either implant or tooth support through geometric analysis. This step was implemented to fully automate the modelling workflow in Grasshopper, eliminating the need for users to manually indicate the type of support. The type of support for the imported FPD was detected using the following steps:

(1) Create a line *L_p_* that connects the centre points, *P_face-g_* and *P_face-o_*, of the gingival face *f_gingival_* and the occlusal face *f_occlusal_*, respectively.
(2) Generate 100 equally spaced planes *E_i_* along the line *L_p_*, each perpendicular to *L_p_*.
(3) Find the intersection curves, *C_i-distal_*, between the planes *E_i_* and the lumen mesh *M_distal_* (analogue *C_i-mesial_* with *M_mesial_* for the mesial side). Retain only the closed curves and remove the initial and final curves to eliminate any errors; see Figure 3d.
(4) Calculate the circularity index (*CI*) of the curves *C_i-distal_* using the formula ${\;CI}_{distal}=\frac{\sum_{0}^{n}\;\frac{4\pi A_{i}}{{Pe}_{i}^{2}}}{n-1}$, where n is the number of curves, *A_i_* represents the area of each curve and *Pe_i_* represents the perimeter of each curve [21].

A circularity value (CI) of 1 indicates a perfect circle. As the value approaches 0, it indicates an irregular shape [21]. For FPDs designed for implant support, the CI is closer to 1, reflecting the abutment’s shape that is closer to a circle (but not a circle), as in the case of tooth-supported FPDs. Conversely, for tooth-supported FPDs, the lumen surface mimics the non-circular shape of a natural tooth. 

In this study, a CI ranging from 0.97 to 1 indicates implant support, while values outside this range refer to tooth support. Both the distal and mesial sides of the implant-supported FPD had a CI of 0.997, indicating a nearly perfect circular shape, whereas the tooth-supported FPD had a CI of 0.922, reflecting the more complex shape of a natural tooth. 

**FPD support generation (step 3)**

In this section, the steps to generate a support for the imported FPD mesh *M_FPD_* are described. Either a tooth support (including a cement layer, abutment tooth and periodontal ligament) or an implant support (cement layer, abutment, implant body and screw) was generated. The algorithm works similarly for both the mesial and distal side; for brevity, only the distal side is addressed here.

**Tooth support**

*Cement Layer*

**Figure 4:**
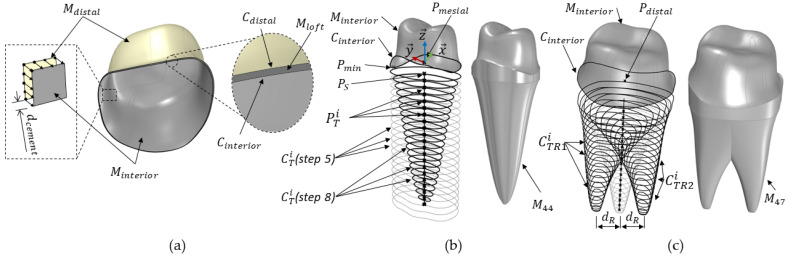
Illustration of the sub-steps to generate the first premolar *M_44_* and second molar *M_47_* abutment tooth support. (**a**) Generation of the cement layer, *M_cement_*. (**b**) Generation of abutment tooth *M_44_*. (**c**) Generation of abutment tooth *M_47_*.

(1) Generate the interior mesh surface, *M_interior_*, by creating a parallel mesh to the distal lumen mesh surface, *M_distal_*, at a distance defined by the cement thickness, *d_cement_*. This involves creating new vertices parallel to each original vertex of *M_distal_* inward along its normal vectors by *d_cement_* and then using these newly calculated vertices to construct *M_interior_*; see Figure 4a.
(2) Extract the boundary curve, *C_interior_*, from *M_interior_*.
(3) Create a lofted mesh surface, *M_loft_*, by interpolating between the curves *C_distal_* and *C_interior_;* see Figure 4a.
(4) Join the surfaces *M_distal_*, *M_loft_* and *M_interior_* to create the geometry of the cement layer, *M_Cement_*.

*Abutment tooth*

The steps required to create the first premolar, which has a single root, are described in this section. Figure 4b illustrates the details of the algorithm.

(1) Divide *C_interior_* into 100 equal-length segments and identify *P_min_* (*x_min_*, *y_min_* and *z_min_*), as the point with the lowest z-coordinate.
(2) Create a point $P_{s}=P_{mesial}-\left|z_{min}\right|\vec{z}$ – ε, where ε prevents the intersection of *C_interior_* with the first projected curve to enable the loft function.
(3) Create a line segment *L_tooth_* defined by the start point *P_s_*, a tangent vector in the $-\vec{z}$ direction and a user-defined length *d_tooth_*.
(4) Divide *L_tooth_* into *i* equally spaced points $P_{T}^{i}$ (with *i* = 20 in this work).
(5) Create *i* planes $\;E_{T}^{i}$ parallel to the *xy*-plane at $\;P_{T}^{i}$ and project *C_interior_* onto each $\;E_{T}^{i}$, yielding curves ${\;C}_{T}^{i}$.
(6) Create Bézier functions *B_x_*(*t*) and *B_y_*(*t*) to scale the curves $C_{T}^{i}$ to mimic the anatomy of a natural tooth, and the non-uniform scaling equation is
  (1) $$B_{x/y}(t)={\left(1-t\right)}^{3}P_{x0/y0}+3{\left(1-t\right)}^{2}tP_{x1/y1}+3{(1-t)t}^{2}P_{x2/y2}+t^{3}P_{x3/y3}$$
  with *P_xi/yi_* as the control points of the Bézier function. The control points used in this study are listed in Table 1. Here, *t* is a parameter ranging from 0 to 1, representing the position along the line *L_tooth_*.

(1) Compute *B_x_*(*t*) and *B_y_*(*t*) for all *t* values, resulting in the arrays *B_x_* and *B_y_*. Each point $P_{T}^{i}\;$ becomes a scaling factor in the x and y directions.
(2) Scale the curves $C_{T}^{i}$ using the non-uniform scaling factors *B_x_* for the *x*-direction and *B_y_* for the *y*-direction.
(3) Create a lofted mesh surface *M_root_* by interpolating through the set of curves $C_{T}^{i}$ and *C_interior_*.
(4) Create a planar surface *M_apex_* from the last curve ${\;C}_{T}^{last}$.
(5) Join *M_interior_*, *M_root_* and *M_apex_* to create the geometry of the first premolar tooth, denoted as *M_44_*.

The steps to create the second molar, which has two roots, are described as follows, with the details of the algorithm illustrated in Figure 4c.

(1) Perform the steps until step 8 (First premolar).
(2) Create a Bézier function *B_T_*(*t*) to translate the scaled curves $C_{T}^{i}$ in both the *x* and *–x* direction to mimic the anatomy of roots, using the control points *P_T_*_0_, *P_T_*_1_, *P_T_*_2_ and *P_T_*_3_, as defined in Table 1.
(3) Compute *B_T_*(*t*) for all *t* values, resulting in the arrays *B_T_*.
(4) Duplicate the curves $C_{T}^{i}$, translating one set in the positive *x*-direction and the other set in the negative *x*-direction by *B_T_ × d_R_*, yielding $C_{TR1}^{i}$ and $C_{TR2}^{i}$. In our work, *d_R_* represents the distance between the furcation and the root apex in the *x*-direction.
(5) Create lofted mesh surfaces *M_root_*_-1_ and *M_root_*_-2_ through the set of curves $C_{TR1}^{i}$ and *C_interior_* and $C_{TR2}^{i}$ and *C_interior_*, respectively.
(6) Create planar surfaces *M_apex_*_-1_ and *M_apex_*_-2_ from the last curves $C_{TR1}^{last}$ and ${\;C}_{TR2}^{last}$, respectively.
(7) Join *M_interior_*, *M_root_*_-1_ and *M_apex_*_-1_ to create the geometry of the first root, denoted as *M_T1_*, and join *M_interior_*, *M_root_*_-2_ and *M_apex_*_-2_ to create the geometry of the second root, denoted as *M_T2_*.
(8) Perform a mesh union on the meshes *M_T_*_1_ and *M_T_*_2_ to create the geometry of the second molar tooth, denoted as *M*_47_.

*Periodontal ligament (PDL)*

Since the steps for generating the periodontal ligament are similar for all teeth, only one example is provided in this section to create the PDL geometry for the first premolar. The following steps were implemented:

(1) Join the mesh surfaces *M_root_* and *M_apex_* to create the surface *M_PDL-in_*, which interfaces with the tooth geometry *M*_44_.
(2) Construct new vertices parallel to each original vertex of *M_PDL-in_* outward along its normal vector by the user-defined periodontal ligament thickness *d_PDL_*. Use these newly created vertices to create *M_PDL-ex_*, which later interfaces with the bone.
(3) Create a lofted mesh surface, *M_PDL-loft_*, using the boundary curves *C_PDL-in_* and *C_PDL-ex_* obtained from *M_PDL-in_* and *M_PDL-ex_*.
(4) Join the surfaces *M_PDL-in_*, *M_PDL-loft_* and *M_PDL-ex_* to form the periodontal ligament geometry *M_PDL_*.
(5) After insertion into bone (step 4), the part of the PDL that was not in contact with the bone was cut.

**Implant support**

In this section, the steps for generating the implant support are described, whereas the whole support consists of a cement layer, abutment, implant body and abutment screw.

*Cement Layer*

The cement layer for the implant support type was generated using the same steps as for tooth support, resulting in the boundary curve *C_interior_* of the interior mesh surface *M_interior_* and the cement layer *M_cement_*.

*Abutment*

(1) Create circles *C_A_*_1_, *C_A_*_2_ and *C_A_*_3_ on planes parallel to the *xy*-plane with user-defined lengths *l_A1_*, *l_A2_* and *l_A3_* and diameters *d_A_*_1_, *d_A_*_2_ and *d_A_*_3_. The plane at the distance *l_A_*_1_ is the implant neck plane *E_implant_* ($\vec{x_{I}},\;\vec{y_{I}}$).
(2) Compute a lofted surface through the set of curves *C_interior_*, *C_A_*_1_, *C_A_*_2_ and *C_A_*_3_. Join this surface with *M_interior_* to create the abutment geometry without a borehole, denoted as *M_A-loft_*.
(3) Create the borehole geometry by creating circles *C_BA_*_1_ and *C_BA_*_2_, each with user defined-lengths *l_BA_*_1_ and *l_BA_*_2_ and diameters *d_BA_*_1_ and *d_BA_*_2_, respectively. Extrude *C_BA_*_1_ upwards to the cement layer and *C_BA_*_2_ downwards to the lower surface of *M_A-loft_*, forming the borehole mesh *M_A-bore_*.
(4) Extract *M_A-bore_* from *M_A-loft_* using a Boolean mesh subtraction operation to create the abutment geometry for implant support, *M_Abutment_*.

*Implant body*

**Figure 5:**
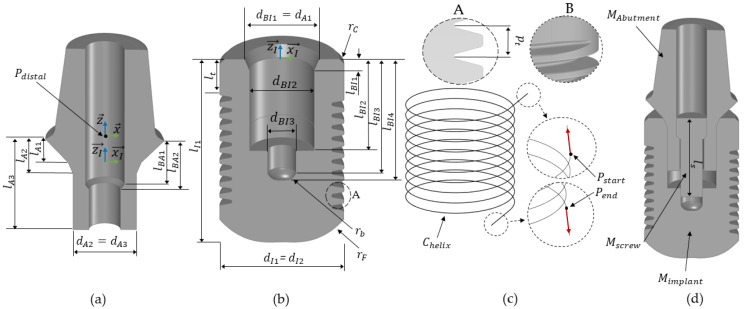
Illustration of the sub-steps involved to generate the implant support for *M_FPD_*. (**a**) Sectional view of the abutment geometry, *M_abutment_*. (**b**) Sectional view of the implant body geometry, *M_implant_*. (**c**) Overview of the helix curve *C_helix_* and the thread profile used in this work. (**d**) Sectional view of the implant support.

(1) Create a cylinder *M_I-loft_* on the plane *E_implant_* with user-defined diameters *d_I_*_1_ (upper diameter) and *d_I2_* (bottom diameter) and length *l_I1_*. In this work, the implant is designed to be straight with *d_I1_* = *d_I_*_2_.
(2) Chamfer the implant body *M_I-loft_* at the upper cylinder edge curve with a radius *r_C_* and fillet the bottom edge of the cylinder with a radius *r_F_*.
(3) Create the borehole geometry *M_I- bore_* using circles with diameters *d_BI1_*(*= d_A_*_1_), *d_BI2_*(*= d_A_*_3_) and *d_BI_*_3_ and lengths *l_BI_*_1_, *l_BI_*_2_, *l_BI_*_3_ and *l_BI_*_4_. For more geometric details, see Figure 5b.
(4) Extract *M_I-bore_* from *M_I-loft_* to create the implant body geometry *M_implant_*.
(5) Construct a helix curve, *C_helix_*, starting *l_t_* below the implant neck plane, with the same diameter *d_I_*_1_ as the implant, a user-defined pitch, *p_t_*, and number of turns, *n_turns_*, along the *z*-axis using the *helix curve* function in Grasshopper.
(6) Extend the curve *C_helix_* by creating tangential segments at its end points, *P_start_* and *P_end_*, to replicate the toolpath movement during the milling process.
(7) Generate the surface of a revolution, *M_thread_*, using the function *sweep rail* with the metric thread profile as the profile curve, the helix curve *C_helix_* as the rail curve and the *z*-axis as the axis of the revolution.
(8) Extract *M_thread_* from the implant body geometry *M_implant_*.

*Abutment screw*

An abutment screw *M_screw_* was generated after the creation of *M_abutment_* and *M_implant_*, with the screw length *l_s_* being the only parameter defined by the user; see Figure 5d.

The values of all user-defined parameters used in this study are listed in Table 1. 

**Table 1:** Geometric parameters and their values used in this study.

Component	User Defined Parameters for Tooth Support, in mm.
Cement layer	*d_cement_ =* 0.1 mm (for mesial and distal side).
Tooth, first premolar	*ε =* 0.15 mm, *d_tooth_ =* 16.3 mm, *P_x_*_0_(0;1), *P_x_*_1_(0.08;0.78), *P_x_*_2_(0.75;0.55), *P_x_*_3_(1;0.05), *P_y_*_0_(0;1), *P_y_*_1_(0.2;0.58), *P_y_*_2_(0.95;0.5), *P_y_*_3_(1;0.05)
Tooth, second molar	*ε =* 0.15 mm, *d_tooth_ =* 12.8 mm, *P_xi_* (same as first premolar), *P_yi_* (same as first premolar), *P_T_*_0_(0;0), *P_T_*_1_(0.35;0.55), *P_T_*_2_(0.4;0.9), *P_T_*_3_(1;1)
PDL	*d_PDL_ =* 0.3 mm (for mesial and distal side)
**Component**	**User Defined Parameters for Implant Support, in mm.**
Cement layer	*d_cement_ =* 0.1 mm
Abutment	*l_A_*_1_ = 1.0 mm, *l_A_*_2_ = 1.5 mm, *l_A_*_3_ = 7.0 mm, *d_A_*_1_ = 3.0 mm, *d_A_*_2_ = 2.6 mm, *d_A_*_3_ = 2.6 mm, *l_BA_*_1_ = 2.0 mm, *l_BA_*_2_ = 2.2 mm, *d_BA_*_1_ = 1.6 mm, *d_BA_*_2_ = 1.3 mm, (mesial and distal side).
Implant body	*d_I_*_1_ = 5.0 mm, *d_I_*_2_ = 5.0 mm, *l_I_*_1_ = 8.0 mm, *r_C_ =* 1.5 mm, *r_F_ =* 0.2 mm, *l_BI_*_1_ = 0.5 mm, *l_BI_*_2_ = 4.0 mm, *l_BI_*_3_ = 5.0 mm, *l_BI_*_4_ = 5.3 mm, *d_BI_*_1_ = 3.0 mm, *d_BI_*_2_ 2.6 = mm, *d_BI_*_3_ = 1.1 mm, *r_b_ =* 0.2 mm, *l_t_ =* 1.5 mm, *p_t_ =* 0.5 mm, *n_turns_ =* 10, (mesial and distal side).
Abutment screw	*l_s_ =* 4.5 mm.

**Insertion of restoration into mandibular bone (step 4)**

**Figure 6:**
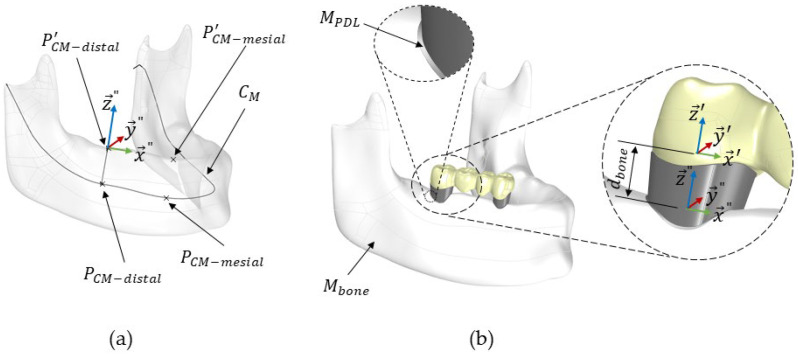
Illustration of the sub-steps to place *M_FPD+S_* in the mandibular bone. (**a**) The local coordinate system *S_bone_*. (**b**) Translation and rotation of *M_support_*.

(1) Merge the FPD (*M_FPD_*) and the support geometries on both the mesial and distal sides. For tooth–tooth support, include *M_cement-distal_*, *M_cement-mesial_*, *M_44_*, *M_47_*, *M_PDL-distal_* and *M_PDL-mesial_*. For implant–implant support, include *M_cement-distal_*, *M_cement-mesial_*, *M_abutment-distal_*, *M_abutment-mesial_*, *M_implant-distal_*, *M_implant-mesial_*, *M_screw-distal_* and *M_screw-mesial_*. This step forms the mesh *M_FPD+S_*.
(2) Define the distal and mesial sides of the FPD, *P_CM-distal_* and *P_CM-mesial_*, on the mandibular centre curve, *C_M_*, as shown in Figure 6a. The distance between these points corresponds to the distance between *P_mesial_* and *P_distal_* of the FPD. Further details about the construction of the centre curve *C_M_* can be found in our previous study [16].
(3) Project the points *P_CM-distal_* and *P_CM-mesial_* onto the crestal mandibular surface, resulting in *P’_CM-distal_* and *P’_CM-mesial_*. The steps required the projection of a point from *C_M_* onto the crestal surface of bone were further explained in a previous work [16].
(4) Define a local coordinate system for the mandibular bone *M_bone_*, denoted as *S_bone_* (${\vec{x}}^{\prime \prime },\;{\vec{y}}^{\prime \prime },\;{\vec{z}}^{\prime \prime }$). This system’s origin is positioned at $P_{CM-distal}^{\prime }$. The *z*-axis (${\vec{z}}^{\prime \prime }$) is aligned with the line connecting *P_CM-distal_* and *P’_CM-distal_*. The *x*-axis (${\vec{x}}^{\prime \prime }$) aligns with the line connecting $P_{CM-distal}^{\prime }$ and ${\;P}_{CM-mesial}^{\prime }$, and the *y*-axis (${\vec{y}}^{\prime \prime }$) is constructed to be orthogonal to both $x^{\prime \prime }$ and ${\;\vec{z}}^{\prime \prime }$; see Figure 6a.
(5) Perform the necessary rotations and translations to align *M_FPD+S_*’s local coordinate system, *S_FPD_* ($\vec{x^{\prime }},\;\vec{y^{\prime }},\;\vec{z^{\prime }}$), precisely with the mandibular bone’s local coordinate system, *S_bone_* ($\vec{x^{\prime \prime }},\vec{y^{\prime \prime }},\vec{z^{\prime \prime }}$), using the Grasshopper functions *Move to point* and *Orient*.
(6) Translate *M_FPD+S_* along the *z*-axis ($\vec{z^{\prime \prime }}$) by a distance *d_bone_*, specified by the user. This distance, *d_bone_* (*d_bone_ = 2.8* mm in this study), represents the gap between *P_distal_* and the crestal surface of the mandibular bone; see Figure 6b.
(7) After *M_FPD+S_* was positioned and aligned, it was extracted from the mandibular geometry, *M_bone_*, using a Boolean mesh subtraction.

**Generation of bone defects (step 5)**

The bone defects were generated using algorithms from our previous work [16]. It has been shown that the shape of bone loss does not affect the stress distribution and that stress is primarily dependent on the depth of bone loss. In the current study, a saucer-shaped defect covering the full buccal–oral width was chosen, and the defects were generated around the implants and the teeth. The bone loss width was set at 10 mm. The sharp corners of the defects were smoothed with a fillet radius of 0.4 mm. In this work, the bone loss depth was varied between 0 mm and 3 mm to study the effect of the support type under different stages of bone loss.

After generation steps 1–5, all geometries were converted to polysurfaces using the function *mesh to polysurface* and were exported to ANSYS for finite element analysis.

### 2.2. Finite Element Analysis

#### 2.2.1. Material Properties, Contact Models and Mesh Size

The proposed parametric algorithm was used to generate geometries with different depths of bone loss for both types of support and different bone qualities. The stress distributions in the periodontal or peri-implant bone and FPD were computed via FEA in ANSYS. The material properties of all components are listed in Table 2. These properties were considered homogeneous, linear and isotropic, except the transition zone of the bone, which had a linear graded Young’s modulus, and the periodontal ligament (PDL), which was defined using a hyper-elastic material model [22]. The first Ogden model defines the behaviour of the PDL using the material parameters *µ_1_*, *α_1_* and *D_1_*. These parameters are $\mu_{1}=0.006\;MPa$, $\alpha_{1}=29.8\;$ and $D_{1}=0.0$, which describe the hyperelastic material properties of the PDL based on the first Ogden model [22]. All surfaces between the different components were defined as bonded.

A graded transition zone between cortical and cancellous bone was modelled. This zone is observed in natural bone and provides a more realistic representation of the bone structure while, at the same time, helps to achieve convergence of the results and to avoid singularities. The transition zone had a thickness of 0.29 mm, and the thickness of the cortical layer was defined according to the bone quality. Misch classified bone quality into four types (D1 to D4) based on the properties of cortical and cancellous bone [30]. The thickness of the cortical bone decreases as follows: 2.5 mm in D1, 2.0 mm in D2, 1.5 mm in D3 and 1.0 mm in D4, while its Young’s modulus $E_{cortical}\;$ remains constant at 13.7 GPa. In contrast, the cancellous bone becomes progressively less dense, with its Young’s modulus $E_{cancellous}$ ranging from 9.5 GPa in D1 to 5.5 GPa in D2, 1.6 GPa in D3 and 0.69 GPa in D4 [29]. The modelling steps of the graded bone approach can be found by Roffmann et al. [28].

The geometries were meshed using quadratic tetrahedral elements due to their complex structure. The mesh was refined in areas of interest, such as the peri-implant interface and the middle connector area. The global mesh size was set to 0.5 mm. The mesh elements of the middle connector were refined to 0.2 mm to achieve convergence [18]. For implant–implant support simulation, the mesh elements of the peri-implant interface were refined to 0.1 mm, while, for tooth–tooth support simulation, it was refined to 0.2 mm. The mesh size of the PDL layer was set to 0.1 mm. The implant–implant support model consisted of approximately 1,949,500 elements and 2,901,700 nodes, while the tooth–tooth support model had 529,000 elements and 837,100 nodes. The overall mesh size, along with the refinement areas and sizes, was determined through a mesh convergence analysis. The convergence was evaluated across different geometries, and the relative errors between successive maximum stress values were carefully evaluated. A mesh quality metric, according to the methodology of Burkhart et al. [31], was performed to ensure high-quality mesh. The criteria required that 95% of the elements have an aspect ratio < 3, a maximum angle deviation within ±70° and a Jacobian ratio > 0.7. The criteria set by Burkhart et al. were fulfilled for all simulations. 

#### 2.2.2. Boundary Conditions and Loading

**Figure 7:**
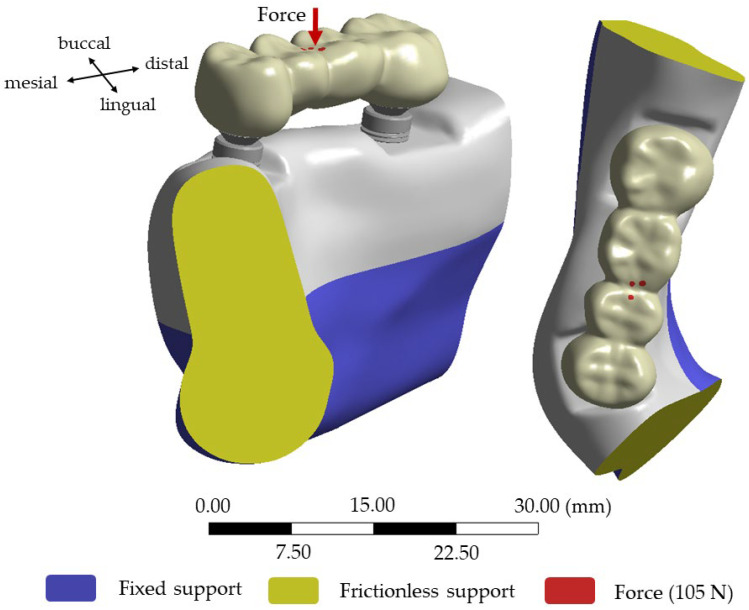
The boundary conditions are shown for the geometry of the implant–implant-supported FPD, with a bone loss of 2 mm.

To reduce the simulation time, the proposed algorithm was applied to a bone segment around the first premolar and second molar. The lower surface of the mandibular bone segment was fixed in three directions (blue surface in Figure 7), and both side surfaces were defined as frictionless (yellow surface in Figure 7). In all simulations, a force of 105 N was applied to 3 small contact areas on the occlusal surface of the FPD (red areas in Figure 7), close to the middle connector and parallel to the implant axis of both implants [20]. Further details about the boundary conditions can be seen in Figure 7. 

#### 2.2.3. Evaluation

The proposed approach for the automatic generation of a support for 4-unit fixed partial dentures was applied for 4-unit FPD, designed to replace teeth between the first premolar and second molar. The support types were analysed under various bone loss defects, ranging from 0 mm to 3 mm, and for different bone qualities, from D1 to D4. The highest maximum principal stresses (tensile stress) in the FPD and bone were computed to evaluate the influence of different support types under different situations of bone defects and bone qualities.

## 3. Results

**Figure 8:**
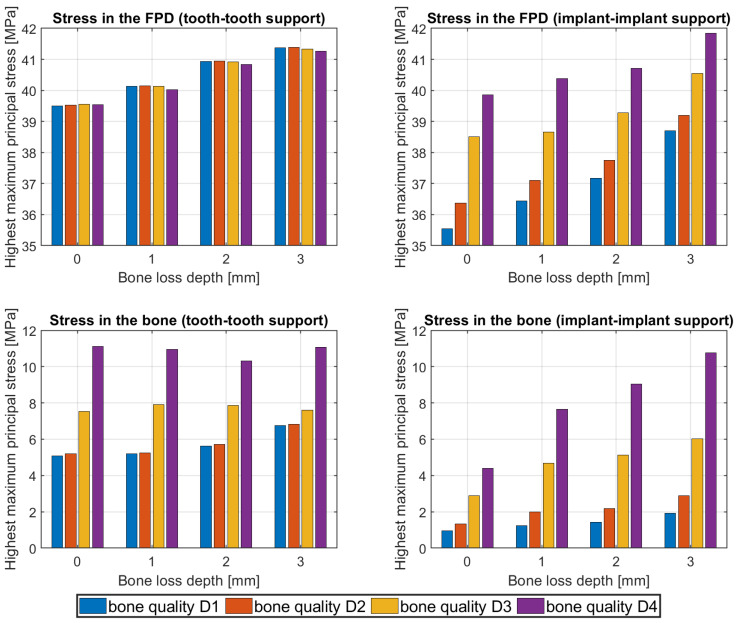
Impact of bone loss depth and bone loss quality on the highest maximum principal stresses in fixed partial denture (FPD) and bone for tooth–tooth (left) and implant–implant (right) support.

In all simulations, the highest maximum principal stress (tensile stress) for the FPD is located on the gingival side of the middle connector. The influence of bone loss and bone quality on the stress peaks in peri-implant bone and the FPD are shown in Figure 8. Increasing the depth of bone loss leads to higher stresses in the FPD for tooth–tooth support, whereas bone quality has no effect on the stress maxima. In contrast, for implant–implant support, higher stress peaks are observed for both increasing the bone loss depth and decreasing the bone quality. 

The stress peaks in the bone are affected by both bone loss depths and bone quality for both types of support. From D1 to D2, the increase is slight, while from D2 to D3 and D3 to D4, the increase in stress is more significant. Another observation is that the stress peaks, observed in the bone for the tooth–tooth support type, are higher than those in implant–implant support. For tooth–tooth support with D1 and D2 bone quality, the stress peaks are located in the cancellous bone at the apices of teeth. For geometries with D3 and D4 bone quality, the stress peaks are located in the cortical bone near the transition zone. For implant–implant geometries, the stress peaks are consistently located in the cortical bone near the transition zone. In all simulations, the stress peaks in the peri-implant bone are observed on the mesial side.

Singularities occurred for geometries with implant–implant support, due to the presence of thin bone components at the crestal bone surface. The region around the singularities was excluded from the analysis.

## 4. Discussion

The aim of this work was to develop an algorithm for the parametric generation of a support for a given 4-unit fixed partial denture. The proposed parametric algorithm was used to investigate the influence of bone loss and bone quality on stresses in FPD and bone for two types of support: implant–implant support and tooth–tooth support.

The goal of developing an algorithm for parametric support generation was successfully achieved for basic support types. This represents one of the necessary steps for the automatic generation of patient–individual CAD-models, which could, for instance, be helpful for a patient-specific finite element analysis-based prognosis once fully developed. While this prognosis goal seems, in principle, aligned with today’s technology level, still, many challenges remain to be overcome. Addressing the support aspect, it would be necessary, for example, to adapt the automated root generation to other clinically observed root types. Moreover, developing a method to automatically extract the relevant patient-specific parameters from existing imaging data would be essential. Regarding the implants, a similar challenge exists, as several implant systems with different geometries exist in the market.

The highest maximum stresses (tensile stresses) in the FPD were located on the gingival side of the middle connector, as was already analysed in deeper detail in a previous study [18]. These stresses increased for progressing bone loss for both support types, as shown in this study. The effect seems slightly stronger for implant support, where the highest maximum stresses at the middle connector increase from 35.6 MPa to 38.7 MPa (8.7%) for D1 bone and from 39.9 MPa to 41.8 MPa (4.8%) for D4 bone when a bone loss of 3 mm was simulated instead of 0 mm. For tooth support, the highest maximum stresses at the middle connector increased from 39.5 MPa to 41.4 MPa (4.8%) for D1 bone and from 39.5 MPa to 41.3 MPa (4.6%) for D4 bone when a bone loss of 3 mm was simulated instead of 0 mm. Overall, the increasing stress originates from the weaker bone support, as the FPD can bend stronger with a less rigid support. One could ask how these findings are quantitatively affected by specific bone design and/or antagonistic teeth. This was not analysed in our study but can be addressed more easily in future studies due to the automation algorithms. In general, too-high stresses should be avoided, as they might lead to material damage in clinical settings.

The bone type affected the FPD stress for about a 4% difference between D1 and D4 in the case of implant support, whereas it does not seem to play a role for tooth support (~0.2% difference). The minimal impact of bone quality on stress distribution of the FPD in tooth–tooth support might be explained by the stiffness of the PDL being relatively low compared to the surrounding bone, which leads to the PDL allowing most of the deformation. In the case of implant support, the implants are stiffer than the surrounding bone, so the bone allows most of the deformation.

The findings regarding the FPD support are in line with the literature from Dittmer et al. and Rand et al. [20,32], where it was shown using finite element analysis that the tensile stresses in the restoration were lower when the FDP support was more rigid. Mahmood et al. [33] found for an experimental 3-unit FPD that a rigid support leads to higher fracture loads. Furthermore, Muddugangadhar et al. conducted a retrospective cohort study on FPDs supported by implants and implant–tooth combinations after 5 years of function. They reported a survival rate of 94.5% for implant-supported FPDs and a lower survival rate of 91.3% for tooth–implant-supported FPDs [12]. 

Regarding the influence of bone quality, Tsouknidas et al. [6] found, for a tooth–implant-supported 3-unit FPD, that the quality of bone and the rigidity of the connection between a natural tooth and an implant influenced both the generated stresses and the displacement of the tooth and the implant. They found that the biggest tooth and implant displacement occurred for type 4 bone and a non-rigid connection.

In this study, simplifications were introduced to reduce the complexity of the model, resulting in inherent limitations. All material properties, with the exception of the PDL and the transition zone, were treated as isotropic, linear and homogeneous. The transition zone had a linear graded Young’s modulus explained in more detail in a previous work [28], and the PDL was modelled using a hyper-elastic material model. PDL fibres, like those incorporated in the approach by Wang et al. [22], were not modelled in this study for complexity reasons. The contact interfaces between the implant body, abutment and abutment screw were considered bonded instead of frictional to reduce the simulation time. A further limitation is that the load was directly applied to the contact points of the occlusal surface rather than using an antagonist, as in a previous study [34]. Additionally, only the bone segment surrounding the FPD was used for the simulations to decrease the number of mesh elements and, consequently, reduce the computational times. Using this segment instead of using the full mandibular, including jaw joints and muscle attachments, might have a noticeable influence on the results. Another limitation of this study is the simplification of the natural shapes of tooth roots, which are missing some anatomical features. Future work should aim for more sophisticated tooth root modelling. Validation using experimental data is also missing currently, as the primary aim of this study was to automate the manual work associated with generating supports for n-unit fixed partial dentures. It is of interest to add this to future works. Furthermore, the mandibular nerve canal was out of the scope of the current work. This geometrical simplification does not affect the overall conclusions and was implemented to reduce the complexity of the 3D model. However, for future individualised patient treatments, the nerve canal should be considered, for example, to automatically generate supports that account for its location and to avoid potential interference. This study used a bone model based on cancellous bone with a homogenised Young’s modulus, simplifying the detailed trabecular structure. While this approach reduces the complexity, it may have an impact on the observed stress distribution.

In this study, only the maximum principal stresses (tensile stresses) were analysed. In the case of bone, compressive stresses are also of interest. Unfortunately, the generated bone loss geometry applied in our model leads to singularities at the crestal bone surface due to the implants threads, as explained by Jemaa et al. [16]. Because of these singularities, these regions were excluded from the analysis. The main advantage of the automated algorithm compared to the semi-automated approach, such as the algorithms developed in our previous work to generate bone loss defects [16], is that it eliminates manual tasks. This improvement not only saves time when generating geometries but also reduces user errors, leading to more consistent and reliable modelling.

In this work, a hyper-elastic modelling approach was chosen for the PDL. In the literature, the PDL has been modelled with various models, such as linear, bilinear, hyper-elastic or viscoelastic behaviour [22,35]. Specifically, if the study focuses on the behaviour of the PDL, the hyper-elastic model provides results that are more accurate [22], although at a higher computational cost. Overall, the finite element simulation time was approximately 25 min per simulation, for each bone quality and bone loss depth, for implant-supported geometry and around 75 min for tooth-supported geometry on a virtual server using 16 CPU cores (Epyc 7713, AMD, Santa Clara, CA, USA) and 256 GB RAM.

The loads applied in this work fall within the range of bite forces for solid food, which Schindler et al. reported to be between 20 N and 120 N [36]. However, masticatory forces can be higher, as reported by Van der Bilt et al., ranging from 306 N ± 42 N to 878 N ± 194 N [37]. While using higher forces would result in higher stress maxima, it would not affect the main conclusions. Furthermore, only one load scenario was tested, but it might be of interest to include further load scenarios in future works.

This study aimed to develop an algorithm for the parametric generation of a support for a given 4-unit fixed partial denture as a preparation for further automation. Although this goal was successfully achieved, further challenges remain for fully automated dental CAD models built for FEA.

## 5. Conclusions

The following conclusions can be drawn from this study:

(1) The proposed algorithm allows an automatic parametrised generation of support for a 4-unit fixed partial denture that can be used to predict the effect of bone loss and bone quality on stresses for patient-specific geometries.
(2) The optimal treatment in terms of support type should be adapted to the patient’s specific needs to ensure long-term stability. Decision support involving automatic modelling, e.g., of support, and simulation might improve optimal treatment from a long-term perspective.
(3) Further work for automated CAD and simulation still remains necessary. For example, the root generation algorithm can still be improved to adapt closer to clinically observed structures.

## Figures and Tables

**Table 2:** Material properties used in the finite element analysis.

Component	Material	Young’s Modulus E [GPa]	Poisson’s Ratio
Implant ^1^	Titanium grade 4	104.5	0.37
Abutment ^1^	Titanium grade 4	104.5	0.37
Implant screw _1_	Titanium grade 5	114.0	0.33
Cement layer ^2^	Glass ionomer cement	15.9	0.33
Abutment tooth ^3^	Polyurethane	3.525	0.33
FPD ^4^	Zirconium dioxide	210	0.27
Cortical bone ^5^	-	13.7	0.3
Transition zone ^6^	-	graded	0.3
Cancellous bone ^7^	-	9.5 (D1 quality)	0.3
	-	5.5 (D2 quality)	0.3
	-	1.6 (D3 quality)	0.3
	-	0.69 (D4 quality)	0.3

^1^ [23], ^2^ [24], ^3^ [25], ^4^ [26], ^5^ [27], ^6^ [28] and ^7^ [29].

## Data Availability

The original contributions presented in the study are included in the article, and further inquiries can be directed to the corresponding author.
